# Targeted and Non-Targeted Metabolomic Evaluation of Cerebrospinal Fluid in Early Phase Schizophrenia: A Pilot Study from the Hopkins First Episode Psychosis Project

**DOI:** 10.3390/metabo15040275

**Published:** 2025-04-15

**Authors:** George E. Jaskiw, Mark E. Obrenovich, Curtis J. Donskey, Farren B. S. Briggs, Sun Sunnie Chung, Anastasiya I. Kalinina, Austin Bolomey, Lindsay N. Hayes, Kun Yang, Robert H. Yolken, Akira Sawa

**Affiliations:** 1 Veterans Affairs Northeast Ohio Healthcare System, Cleveland, OH 44106, USA; dr.obrenovich@gmail.com (M.E.O.); curtis.donskey@va.gov (C.J.D.); austinbolomey@gmail.com (A.B.); 2 School of Medicine, Case Western Reserve University, Cleveland, OH 44106, USA; 3 Department of Chemistry, Case Western Reserve University, Cleveland, OH 44106, USA; 4 Department of Medicinal and Biological Chemistry, University of Toledo, Toledo, OH 43606, USA; 5 Department Public Health Sciences, Miller School of Medicine, University of Miami, Miami, FL 33136, USA; fbb31@med.miami.edu; 6 Department of Computer Science, Cleveland State University, Cleveland, OH 44115, USA; s.chung@csuohio.edu (S.S.C.); a.kalinina@alumni.csuohio.edu (A.I.K.); 7 Department of Neuroscience, Johns Hopkins University School of Medicine, Baltimore, MD 21205, USA; lindsay-hayes@ouhsc.edu; 8 Department of Cell Biology, University of Oklahoma Health Sciences Center, Oklahoma City, OK 73104, USA; 9 Department of Psychiatry and Behavioral Sciences, Johns Hopkins University School of Medicine, Baltimore, MD 21287, USA; kunyang@jhmi.edu; 10 Stanley Division of Developmental Neurovirology, Johns Hopkins School of Medicine, The Johns Hopkins Hospital, Baltimore, MD 21287, USA; rhyolken@gmail.com; 11 Departments of Psychiatry, Neuroscience, Biomedical Engineering, Pharmacology, Genetic Medicine, Johns Hopkins University School of Medicine, Baltimore, MD 21287, USA; 12 Department of Mental Health, Johns Hopkins University Bloomberg School of Public Health, Baltimore, MD 21205, USA

**Keywords:** metabolome, cerebrospinal fluid, first-episode psychosis, schizophrenia, biomarker, gut microbiome, targeted, non-targeted

## Abstract

(1) Background: The lack of reliable biomarkers remains a significant barrier to improving outcomes for patients with schizophrenia. While metabolomic analyses of blood, urine, and feces have been explored, results have been inconsistent. Compared to peripheral compartments, cerebrospinal fluid (CSF) more closely reflects the chemical composition of brain extracellular fluid. Given that brain dysregulation may be more pronounced during the first episode of psychosis (FEP), we hypothesized that metabolomic analysis of CSF from FEP patients could reveal disease-associated biomarkers. (2) Methods: We recruited 15 patients within 24 months of psychosis onset (DSM-4 criteria) and 14 control participants through the Johns Hopkins Schizophrenia Center. CSF samples were analyzed using both non-targeted and targeted liquid chromatography–mass spectrometry. (3) Results: The non-targeted analysis identified lower levels of N-acetylneuraminic acid and N-acetyl-L-aspartic acid in the FEP group, while levels of uric acid were elevated. The targeted analysis focused on indolic and phenolic molecules previously linked to neuropsychiatric disorders. Notably, L-phenylalanine and 4-hydroxycinnamic acid levels were lower in the FEP group, and this difference remained significant after adjusting for age and sex. However, none of the significant differences in analyte levels between the groups survived an adjustment for multiple comparisons. (4) Conclusions: Our intriguing but preliminary associations align with results from other investigational approaches and highlight potential CSF analytes that warrant further study in larger samples.

## 1. Introduction

Schizophrenia is a chronic brain disorder characterized by both phenotypic and neurobiological heterogeneity, often leading to severe psychosocial impairment [1]. Efforts to improve patient outcomes have been hindered by a fundamental challenge: diagnosis and monitoring rely on variable and non-specific clinical features, such as symptomatology and disease course [1]. The extent to which these phenotypic similarities reflect meaningful neurobiological indices remains unclear. Currently, no reliable biological markers exist to guide diagnosis, identify underlying pathophysiology, or personalize treatment [2]. Developing such biomarkers is a critical unmet need—not only in schizophrenia research but across the field of psychiatry [3].

The current consensus holds that no single phenomenological or neurobiological measure will be specific to schizophrenia [4,5,6]. Instead, future biomarkers will likely emerge from a combination of factors across multiple modalities, including genetics, proteomics, metabolomics, imaging, and cognitive assessments [6,7]. Our research has focused on the metabolome [8,9,10,11,12], the vast collection of small molecules (MW < 2000 Da) present in biological systems [13]. These molecules can be generated by endogenous host pathways [13], by gut microbiota (GMB), or co-metabolic interactions between the two [14]. Studies of peripheral compartments such as blood, urine, and feces have thus far produced heterogeneous findings with limited reproducibility [15,16,17]. While methodological differences undoubtedly contribute, a major challenge is that peripheral metabolomes are sensitive to numerous influences outside the central nervous system and are characterized by very high data variance [18,19].

Cerebrospinal fluid (CSF) offers distinct advantages as a source of potential biomarkers. Compared to other biofluids, it more closely approximates the chemical composition of the brain [20,21]. The blood–brain (BBB) and blood–CSF barriers restrict peripheral influences on CSF composition [21]. As a result, the variance in metabolomic data derived from the CSF is usually less than that in peripheral compartments [22]. Elements of the CSF metabolome, however, still remain vulnerable to effects of antipsychotic drug (APD) treatment [23,24], substance use [25,26], and medical comorbidities like Type 2 diabetes mellitus [27]. The impact of such factors likely increases with the duration of exposure. In contrast, neurobiological changes identified during the early course of schizophrenia—known as the first episode of psychosis (FEP)—are more likely to reflect disorder-specific mechanisms. Our previous research highlighted that certain metabolites, particularly those with indolic or phenolic structures produced partially by the gut microbiota (GMB), are recurrently implicated in schizophrenia, marking them as promising research targets [8]. To further explore this, we conducted a pilot study comparing FEP patients and healthy controls (HCs), using both non-targeted and targeted metabolomic analyses with a focus on GMB-dependent small molecules derived from aromatic amino acids.

## 2. Materials and Methods

### 2.1. Participants

Participants were recruited through the Johns Hopkins Schizophrenia Center as part of its ongoing FEP research initiative [28,29]. The study was approved by the Institutional Review Board (IRB) and conducted in accordance with the World Medical Association’s Code of Ethics. Written informed consent was obtained from all participants. Participants consented to undergo laboratory testing and a lumbar puncture, with the required laboratory tests, including aPTT and PT/INR, completed within one month of each procedure. They also had to abstain from all non-steroidal anti-inflammatory drugs (NSAIDs), such as Ibuprofen (Advil, Motrin), Aleve (Naproxen), Aspirin, and Excedrin, for at least 10 days prior to the lumbar puncture. Individuals with active substance abuse in the preceding two months, including alcohol, prescribed stimulants, opioids, or illicit substances (excluding cannabis and synthetic cannabinoid receptor agonists), were excluded. Nicotine use was permitted. Additionally, participants with a bleeding or clotting disorder, as determined by a study team member upon reviewing laboratory test results, or those who failed to complete the necessary laboratory tests preceding the lumbar puncture, were deemed ineligible for participation.

Patients were diagnosed with schizophrenia based on medical records and a structured clinical interview (Structured Clinical Interview for DSM-IV, 2004) and were 18–35 years old at the time of recruitment. CSF samples were collected 9:00–15:00H without regard to nutritional status. Healthy controls (HCs) were age- and sex-matched, with no history of traumatic brain injury, cancer, abnormal bleeding, serious viral infections (e.g., HIV, hepatitis), or neurological or psychiatric disorders.

All CSF samples were immediately frozen after collection and maintained at −80 °C. CSF aliquots (100 μL) were coded and supplied to the principal investigator (G.E.J.) along with data on participant’s age, gender, race and group classification, but blinded as to FEP or HC status.

### 2.2. Metabolomic Analyses

#### 2.2.1. Chemicals and Reagents

All chemicals and reagents were of the highest purity and grade commercially available. Authentic standards were purchased from Sigma-Aldrich^®^ (St. Louis, MO, USA), Toronto Research Chemicals^®^ (Toronto, ON, Canada) or other sources [10]. LCMS grade solvents: water; acetonitrile; formic acid; and methanol were purchased from Fisher Scientific (Pittsburgh, PA, USA).

#### 2.2.2. Sample Preparation

Metabolomic analyses were conducted within the Proteomics and Metabolomics Core of the Lerner Research Institute at the Cleveland Clinic Foundation (Cleveland, OH), without information on the diagnostic assignment of the groups. Each frozen CSF sample was thawed, vortexed for 1 min, and then centrifuged (14,000× *g* at 8 °C × 10 min). No attempt was made to precipitate proteins, given their very low levels in CSF [21]. A total of 100 μL of resultant supernatant was aliquoted into a clean Eppendorf tube, to which was added 10 μL of internal standard mixture specific for either the targeted or non-targeted analysis (Supplementary Table S1). Then, 50 μL aliquots were assigned for non-targeted and targeted analysis, respectively.

#### 2.2.3. Non-Targeted Analysis

Samples were diluted 1:5 in chilled methanol containing internal standards (Supplementary Table S2). One-microliter aliquots taken from each sample were pooled and this quality control (QC) standard was analyzed every 10 injections.

A total of 5 μL of each sample was injected onto a 10 cm C18 column (Thermo Fisher, San Jose, CA, USA) coupled to a Vanquish UHPLC running at 0.25 mL/min using water and 0.1%formic acid as solvent A and acetonitrile and 0.1% formic acid as solvent B (Supplementary Table S3). The Orbitrap Q Exactive HF was operated in positive and negative electrospray ionization modes in different LC-MS runs over a mass range of 56–850 Da using full MS at 120,000 resolution. Data-dependent acquisitions (DDAs) were obtained on the pooled QC sample. The DDAs MS full scans at a resolution of 120,000 and higher-energy collisional dissociation (HCD) MS/MS scans taken on the top 10 most abundant ions at a resolution of 30,000 with dynamic exclusion of 40 s and the apex trigger set from 2.0 to 4.0 s. The resolution of the MS2 scans was taken at a stepped normalized collision energy (NCE) of 20.0, 30.0, and 45.0.

Data were processed using MSDIAL [30] (v.4.92) for feature detection, identification, and alignment using parameters optimized for data acquired on an Orbitrap mass spectrometer. MS1 and MS2 were set to profile mode in both positive and negative ionization modes. Peak detection of MS1 and MS2 spectra were set to tolerances of 0.01 Da and 0.025 Da, respectively, over a mass range of 56–850 m/z with minimum peak width and height of 5 and 1,000,000. Annotation was performed using publicly available libraries from MassBank of North America (MoNA) [31] containing 13,303 unique compounds (positive mode) 2 and MSDIAL Metabolomics MSP Spectral Kit 3 [30] containing 12,879 unique compounds (negative mode) with an 80% identification cut-off score.

Spectral features from MSDIAL processed data were further analyzed via MetaboAnalyst 5.0 [32]. Features with >80% non-zero values were excluded. Remaining zero/missing values were replaced by one-fifth of the minimum peak height over all samples, subjected to sum normalization, log-transformed, and autoscaled prior to downstream statistical analysis. A raw *p*-value less than 0.05 was chosen for significance. All *p*-values were corrected via the Benjamini–Hochberg procedure for false discovery rate (FDR) with a threshold of 0.05. Fold change analysis was performed. Multivariate principal component analysis (PCA) and hierarchical clustering were performed for understanding metabolite variation and expression patterns between groups. Statistical analysis was performed by comparing the FEP group to controls. All metabolites that statistically differed between the cases and control were identified based on MS1-level matches to the spectral library. The diagnostic group assignment was provided to the principal investigator (G.E.J.) after the data were generated and shared with the Johns Hopkins Schizophrenia Center (R.Y.K, A.S.).

Pathway enrichment analysis was performed to identify top 25 altered pathways for putatively identified metabolites in the dataset. In this analysis, the pathway-associated metabolite sets the Small Molecule Pathway Database 5 (SMPDB) [33], which consists of 99 pathways and which was used to map the putatively identified ions to various pathways.

#### 2.2.4. Targeted Analysis

We used authentic standards with an emphasis on high-value molecules derived from aromatic amino acids and generated in whole or in part by the GMB [8]. The internal standard was phenylalanine-d5. Samples were injected onto an F5 column (Phenomenex) coupled with MS/MS (TSQ Quantiva, Thermo Fisher/Vanquish, Thermos Fisher UPHLC.

#### 2.2.5. Preparation of Standards

A standard stock solution containing all the compounds of interest as well as the internal standards (1 mg/mL) was prepared in a suitable solvent (LC/MS grade water or methanol), initially blanketed with nitrogen gas, in glass vials equipped with Teflon^®^-lined screw caps and then stored at –20 °C. The thawed stock solution was serially diluted to obtain work solutions in the range of 10–1000 ng/mL to support the generation of standard curves for quantitation of the metabolites. For each metabolite, aliquots (10–10,000 ng/mL) were prepared and treated as samples by adding an internal standard to each vial.

#### 2.2.6. LC/MS/MS Analysis

The LC/MS/MS analysis of targeted metabolites was carried out using a triple quadrupole tandem mass spectrometer (TSQ Quantiva, Thermo Fisher Scientific, Waltham, MA, USA) equipped with an electrospray ionization (ESI) interface. The mass spectrometer was coupled to the outlet of HPLC system that consisted of an UHPLC system (Vanquish, Thermo Fisher Scientific, Waltham, MA, USA), including an auto sampler with refrigerated sample compartment and inline vacuum degasser. In brief, 5 μL of extracted sample was injected on a C18 column (Luna, 2.6 µm, 150 x 2 mm, Phenomenex, Torrance, CA, USA) with the flow rate of 0.2 mL/min at 25 C (mobile phases A-water, B-acetonitrile. Mobile phase B was 0% 0–2 min, a linear gradient 0–100% B at 2–8 min, 100% 8–16 min, linear gradient 100–0% 16–16.1 min, and then kept at 0% B for 8 min). The ESI mass spectrometric detection was performed in both the positive and negative ionization modes, with an ion spray voltage at 2.5 kV, sheath gas at 35 Arb, and Aux gas at 20 Arb. The ion transfer tube and vaporizer temperatures were set at 350 °C and 250 °C, respectively. The quantitative analysis was performed using Selected Reaction Monitoring (SRM) for all the metabolites. Ultrapure argon (99.99%) was used as a collision gas at the pressure of 2 millitorr for collision-induced dissociation. Individual standard calibration curves for each compound were used to calculate the concentration of metabolites in samples as described relative to internal standards [10]. For normalization, we used the peak area ratio (internal standard/standards) in the calibration curve. The peak area for each compound was manually integrated using ThermoFisher Tracefinder ^®^ 4.1.

#### 2.2.7. Data Processing

Data were processed by MetaboAnalyst 6.0 [33]. Characteristics with at least one detected sample per group were used for further statistical analysis. Zero/missing values were replaced by one-fifth of the minimum peak height for each feature, which was normalized by sum, log-transformed and autoscaled. Individual statistical comparisons were conducted by Wilcoxon with a *p* < 0.05 and a false-discovery rate (FDR) < 0.05. Fold change (FC) was calculated. Random forest, a machine learning approach, was used to identify features most predictive of FEP.

## 3. Results

### 3.1. Participant Sample

The original dataset had been designed with a 1:1 match, n = 15 FEP, and n = 15 HC. After the chemical analyses had been completed, we learned that one of the HCs had been hospitalized psychiatrically. Accordingly, that control was removed, and statistical analyses conducted on the truncated dataset of n = 15 FEP and n = 14 HC (Supplementary Table S3).

FEP and HC participants did not differ demographically (Table 1 and Supplementary Table S3). There was a weak trend (*p* < 0.01) for FEP samples to have a shorter freezer time than those from the HCs. The characteristics of the FEP group were, 12/15 known to be on APDs, n = 3 schizoaffective/n = 12 schizophrenia, age at illness onset 22.64 ± 1.18 y, duration of illness 1.19 ± 0.26 y (Supplementary Table S3).

Nicotine use history and cannabis use history was available for n = 12/15 FEP and n = 12/14 HC. The proportion of known nicotine users in the FEP (5/12) trended (χ^2^ < 0.06) to be higher than in the HC group (1/12) (Supplementary Table S3). Cannabis use in the two months preceding enrollment was an exclusionary criterion. Nonetheless, one HC and two FEP participants endorsed cannabis use (Supplementary Table S3).

### 3.2. Non-Targeted Data

Non-targeted C18 metabolomics data analysis identified a total of 7196 metabolite features with 1449 MS1-level matches and 22 MS2-level matched to MSP spectral databases (Table 2). Data quality control was assessed using CV statistics computed for each metabolite in the pooled QC samples, and most of these had a CV of less than 30%.

**Figure 1:**
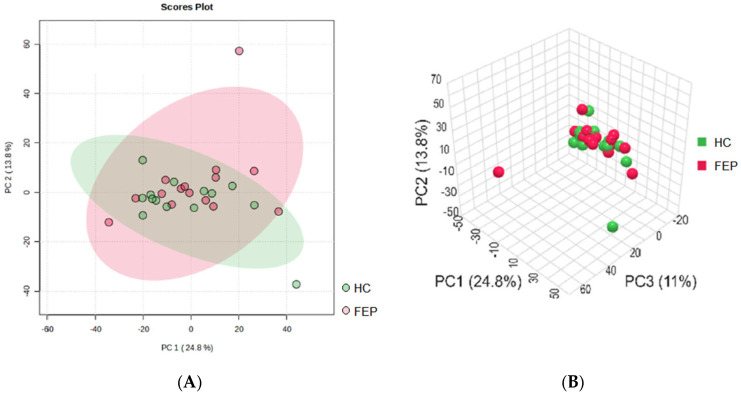
Non-targeted data for healthy controls (HCs) v first-episode psychosis (FEP) as evaluated by (**A**) unsupervised multivariate PCA, (**B**) supervised multivariate OPSL-DA.

**Figure 2:**
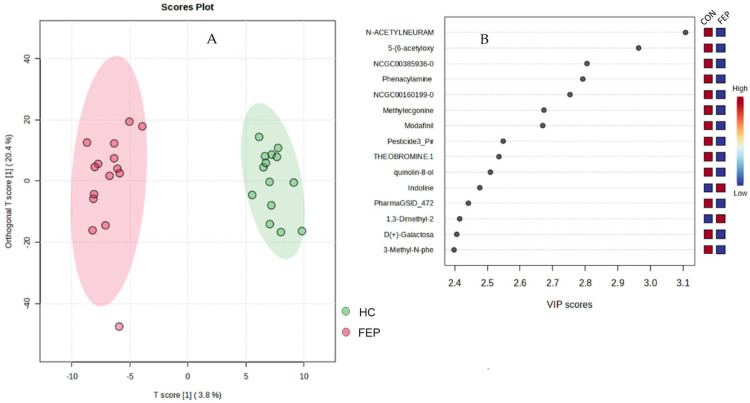
Non-targeted data for healthy controls (HCs) v first-episode psychosis (FEP) (**A**) evaluated by supervised multivariate OPSL-DA, (**B**) showing features with 15 highest VIP scores.

**Figure 3:**
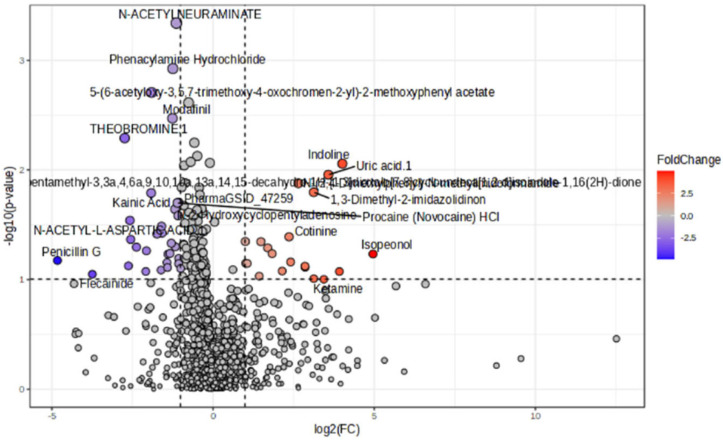
Volcano plot of the non-targeted features.

**Figure 4:**
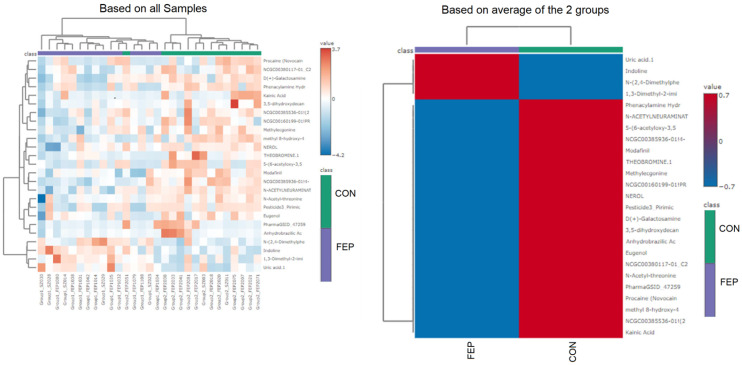
Heatmap of the top 25 features generated by the non-targeted analysis.

Metabolite differences between the HC and FEP groups were revealed by unsupervised multivariate PCA (Figure 1) as well as by supervised multivariate OPSL-DA (Figure 2A) analysis. While PCA analysis showed poor separation, this was maximized by OPLSD-DA analysis, as demonstrated by clear cluster between groups in the OPLS-DA score plots, and the top 15 metabolites with highest VIP scores were reported (Figure 2B and Supplementary Table S4). Examination of FC for metabolites in FEP v HCs by *t*-tests showed n = 23 metabolites that reached significance at raw *p*-value (Table 3 and Table 4, Figure 3). A total of n = 8 small molecules (N-Acetylneuraminate, Phenacylamine Hydrochloride, 5-(6-acetyloxy-3,5,7-trimethoxy-4-oxochromen-2-yl)-2-methoxyphenyl acetate, Modafinil, Theobromine, Indoline, 1,3-Dimethyl-2-imidazolidinon, PharmaGSID_47259) were among the top 15 identified both by VIP scores and by *t*-tests of FC (Table 5). After correction for multiple testing, none of the pairwise comparisons remained significant (FDR < 0.05). The top 25 features are evident in the heat map (Figure 4).

### 3.3. Targeted Data

**Figure 5:**
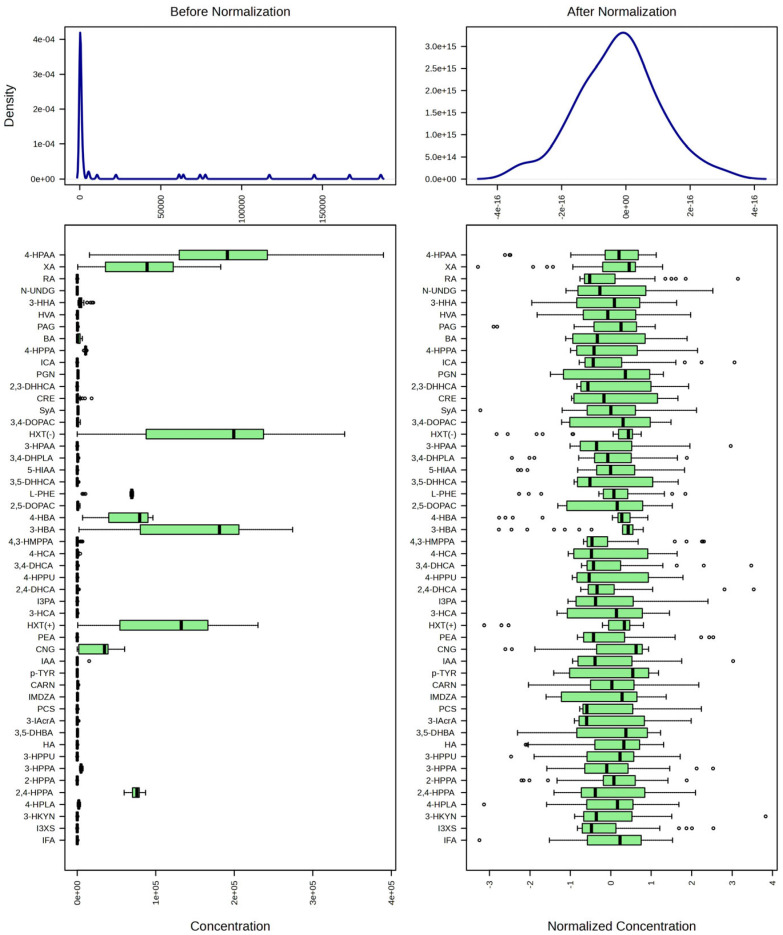
The distribution of targeted features in which at least one value was present. Zero/missing values were replaced by one-fifth of the minimum peak height for each feature. The data were log-transformed (base 10), sum-normalized, and auto-scaled.

The targeted analysis identified 54 compounds, including nine previously unreported in CSF (NPID) (Figure 5, Table 5). This work was conducted during the early months of the COVID-19 pandemic, when laboratory access restrictions prevented us from following rigorous quality control measures. Consequently, we did not confirm adequate chromatographic separation of 10 select standards or generate calibration curves with perfect linearity for these 10 compounds, which we classified as “identified but not quantified” (IDNQ). Thus, we conducted statistical analysis only on compounds with linearity ranges corresponding to our resulting values. Several targeted isomers with a molecular weight of 182.17 exhibited poorer separation than expected. Hence, we were unable to definitively identify and quantify 2,4-DHHCA, 3,4-DHHCA, and 3,3-HPHPA.

**Figure 6:**
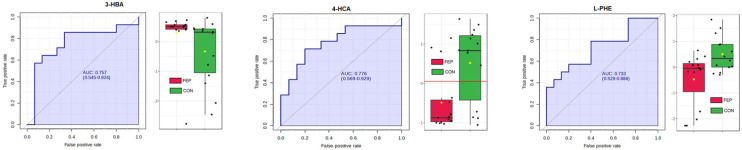
Three characteristics from the targeted analysis (3-hydroxybenzoic acid (3-HBA), 4-hydroxycinnamic acid (4-HCA) phenylalanine (positive mode) (L-PHE(+)) were significantly different (Wilcoxon, *p* < 0.05) between controls (CON) and first-episode psychosis (FEP) based on unadjusted data. 4-HCA and PHE(+) retained significance after adjustment for sex, race, and gender. None reached an FDR < 0.1. Area under the curve (AUC) with confidence interval.

Statistical analyses were conducted on transformed data. Levels of 3 compounds (4-HCA, 3-HBA, PHE(+))—differed significantly (Wilcoxon, *p* < 0.05) between the HC and FEP groups (Table 6). Among them, PHE(+) and 4-HCA retained significance after adjusting for age, sex, and race, with AUC values > 0.7 (Figure 6). No characteristics met an FDR < 0.1 threshold.

## 4. Discussion

This was an exploratory study with a relatively small sample size. While the FEP and HC groups were matched for gender, race, and age (Table 1), there was a slight imbalance in participant numbers due to the exclusion of one HC who was later psychiatrically hospitalized. However, both groups were drawn from a well-established, specialized FEP program, which has previously reported multiple biological differences between FEP and HC cohorts [28,29].

The non-targeted analysis yielded an 18% feature matching rate (Table 2), consistent with expectations for this approach. Principal component analysis (PCA) plots, visualized in both two- and three-dimensional views, showed weak cluster separation between the groups (Figure 1). As anticipated, separation improved under a supervised OPLS-DA model (Figure 2). Several of the top 15 small molecules identified as significantly different between the groups based on fold change (FC) and *t*-tests (Table 4) deserve further consideration, even though the significant differences did not survive an adjustment for multiple comparisons.

N-acetylneuraminic acid (sialic acid) (Neu5Ac) is the acylated derivative of neuraminic acid and serves as a common terminal component of many glycoproteins and glycolipids [34]. Neu5Ac is located predominantly in neurons and is the second most abundant amino acid in the human CNS after glutamate [35]. Homopolymers of Neu5Ac form polysialic acid (PSA), which interacts with the neural cell adhesion molecule (NCAM). Lower levels of PSA-NCAM have been reported in the entorhinal cortex of patients with Alzheimer’s Disease and Parkinson’s Disease and correlate inversely with hyperphosphorylated tau accumulation [36]. PSA-NCAMs are also downregulated in brain regions in schizophrenia [37]. Lower CSF levels of Neu5Ac in schizophrenia were first reported over 65 years ago [38] but the finding was inconsistently replicated in early studies [39,40]. A more recent targeted study found lower levels in frontal cortex tissue of patients with schizophrenia [41].

Brain levels of Neu5Ac are readily measured in vivo by magnetic resonance spectroscopy (MRS) [42]. Brain NAcNeu is recognized as a general marker for neuronal health [42] and cognitive function [43]. Consistently lower tissue levels of NAcNeu have been reported across multiple brain regions in both first-episode psychosis (FEP) and chronic schizophrenia (SCZ) patients. Notably, lower NAcNeu levels in the anterior cingulate cortex and thalamus, as detected by MRS, were associated with psychotic relapse in an FEP subgroup from the Johns Hopkins program [28]. Additionally, a recent large-scale meta-analysis identified lower frontal Neu5A as one of the few Class I diagnostic biomarkers for schizophrenia [2]. Our study is the first to extend this association to Neu5A levels in CSF (Table 4). Blood NAcNeu levels do not correlate with brain levels and show no clear associations with cognition or overall brain integrity [44].

Neu5Ac can affect the regulation of a host of voltage-gated potassium and sodium channels [45]; as a group, these have been implicated in schizophrenia [46]. Additionally, interactions of homopolymers of Neu5Ac with NCAM (NCAM) play a critical role in neural cell functions, including migration, axon and dendrite growth, remodeling, and neuroplasticity [36,47]. Impaired neuroplasticity, in particular, may underlie the negative symptoms and cognitive deficits of schizophrenia [48].

Uric acid (UA) is a well-known radical scavenger with antioxidant actions [49]. Compared to HCs, FEP patients with FEP have frequently, but not universally, been reported to have lower serum levels of UA [50,51,52,53]. Potential covariates influencing UA levels include APD status, age, gender, and BMI [52,54,55,56]. Several older studies identified a weak positive correlation between CSF and serum UA levels in patients with schizophrenia or psychosis [57,58]. However, no significant difference in CSF UA levels has been reported between healthy controls and individuals with chronic schizophrenia [58,59]. To our knowledge, no prior studies have directly compared CSF UA levels between FEP patients and controls. Our finding of elevated CSF UA levels in FEP (Table 4) may reflect increased nucleic acid metabolism driven by the pro-inflammatory and oxidative stress states implicated in early psychosis [50,60,61,62]. On the other hand, given the 2:1 lumbar:ventricular gradient for CSF UA [63] and a > 50:1 ratio for serum:CSF UA [64], the increased BBB permeability associated with FEP [65] may also contribute.

The elevated CSF cotinine levels observed in our FEP sample (Table 4) are consistent with cotinine being a well-established biomarker of nicotine consumption [66]. Once in circulation, cotinine readily crosses the blood–brain barrier (BBB) and blood–CSF barrier, where it serves as an index of nicotine exposure [67]. Nicotine use is highly prevalent in patients with schizophrenia from the prodromal stage onwards [68,69]. This likely explains lower CSF theophylline and theobromine levels in the FEP group (Table 4). Cigarette smoking increases metabolism and plasma clearance of these caffeine metabolites [70], both of which readily cross the BBB [71]. Schizophrenia is associated with elevated levels of coffee consumption [72,73]. The resulting induction of upregulated theophylline clearance [70] would explain the lower fasting theophylline levels.

Several other characteristics also exhibited relatively large fold changes (FC) (Table 4). Certain indoline derivatives, known for their antioxidant, anti-inflammatory, and serotonergic properties, are capable of crossing the blood–brain barrier (BBB) [74,75,76]. N-(2,4-dimethylphenyl) formamide, a neurotoxin that can be generated by certain bacteria [77] or from the breakdown of pesticides [78], was putatively identified. None of these compounds have been previously implicated in neuropsychiatric conditions.

We conducted the targeted analysis when pandemic-related occupational restrictions did not allow us to follow our usual quality monitoring processes. As a result, we were unable to definitively identify three structural isomers of MW 182.17 (2,4-DHHCA, 3,4-DHHCA, 3-HPHPA), which we previously quantified in human CSF [10]. We were, however, able to identify and quantify seven additional compounds which had not been previously reported in human CSF, including 3,4-DHPLA (3-(3,4-dihydroxyphenyl)-2-hydroxypropanoic acid), PGN (benzene-1,3,5-triol), 4,3-HMPPA (3-(4-hydroxy-3-methoxyphenyl)propanoic acid), CNG (2-[[(E)-3-phenylprop-2-enoyl]amino]acetic acid), 2,4-HPPA (2-(4-hydroxyphenyl)propanoic acid), 3,4-HPPA (3-(4-hydroxyphenyl)propanoic acid), and 4-EPS (4-ethylphenyl) hydrogen sulfate) (Table 5).

Missing data are a common challenge in metabolomic investigations, and while their occurrence is typically non-random, the method chosen to handle them can significantly influence the outcome of the analysis The widely used threshold of 60–80% zero values to exclude variables from further analysis risks conflating compounds that are truly absent with those simply below the detection limit [79]. In our pilot study, we chose to include all compounds with at least one non-zero value in both groups. Interestingly, it was found that two-thirds of the characteristics for which group differences trended toward significance had non-missing values greater than 85% (Table 6). L-PHE and 4-HCA were the two molecules that reached a traditional level of significance (*p* < 0.05) after adjusting for age, gender, and race (Table 6), but not after a correction for multiple comparisons.

L-PHE levels were lower in the CSF of the FEP group compared to the HC group (Figure 6). A previous small sample study reported elevated L-PHE levels in the CSF of mostly medicated patients with chronic schizophrenia, though dietary status was not specified [80]. It is generally accepted that CSF levels of L-PHE are primarily influenced by the competitive transport of large neutral amino acids from plasma across the BBB [81]. In our prior research, we were unable to demonstrate such regulation in fasted vervet monkeys or humans but did confirm a positive correlation between serum and CSF L-PHE levels [82]. Given that we did not measure concurrent blood levels, our current study offers no information on the BBB dysfunction reported in other FEP studies [65].

Several early small-sample studies found no difference in plasma L-PHE levels between patients with chronic schizophrenia and controls, regardless of antipsychotic drug (APD) status [83,84]. However, one study reported elevated plasma L-PHE levels and an increased serum L-PHE/L-tyrosine (L-TYR) ratio in a large sample of patients with schizophrenia, despite normal L-TYR levels. These patients were treated with APDs, though the duration of the disease and fasting status at the time of sampling were not specified [85]. Similar serum abnormalities were observed in cases of anti-NMDAR encephalitis and associated with neuropsychiatric symptoms [86]. Altered systemic L-PHE metabolism in chronic schizophrenia has been documented and confirmed by administering radiolabeled L-PHE and then measuring radiolabel in exhaled air [87,88]. Differences in patient selection, medication, and dietary status likely contribute to the variability in findings.

In the CNS, L-PHE can be converted successively to L-TYR, L-dihydroxyphenylalanine (L-DOPA) and dopamine, the neurotransmitter most strongly implicated in the pathophysiology of schizophrenia [89]. Lower levels of brain L-PHE could reflect an altered balance that favors L-TYR and in turn dopamine synthesis. Elevated mesostriatal DA-mediated transmission is thought to contribute to the pathogenesis and maintenance of positive symptoms of schizophrenia [90].

4-HCA is a phenolic acid synthesized from L-PHE or L-TYR via the shikimate pathway which is present in microbes, mushrooms, and plants but not mammals [13,91,92]. However, the human GMB can release and/or synthesize 4-HCA from dietary sources [93,94,95]. Therefore, any 4-HCA in human CSF must be derived from diet and/or the GMB. Whether the lower 4-HCA levels in FEP can be attributed to differences in diet, the GMB or host metabolism remains to be determined. In vitro, 4-HCA lowers intracellular levels of the antioxidant UA by promoting its excretion. Indeed, in the current study, lower 4-HCA levels (Figure 6) were observed alongside elevated UA levels (Table 4). Overall, our CSF levels of 4-HCA were within the range of their reported in vitro bioactivity in human cells [9] including in their ability to lower UA.

4-HCA is also bioactive in vivo in rodent models [96]. It generally displays anti-inflammatory, antioxidant, and neuroprotective effects [96,97,98]. Thus, the lower CSF 4-HCA levels in FEP (Figure 6) may reflect a lower capacity to mitigate inflammatory processes implicated in FEP [62].

We also identified and quantified seven phenolic compounds which had not been previously reported in human CSF 3,4-DHPLA (3-(3,4-dihydroxyphenyl)-2-hydroxypropanoic acid), PGN (benzene-1,3,5-triol), 4,3-HMPPA (3-(4-hydroxy-3-methoxyphenyl)propanoic acid), CNG (2-[[(E)-3-phenylprop-2-enoyl]amino]acetic acid), 2,4-HPPA (2-(4-hydroxyphenyl)propanoic acid), 4-HPPA (3-(4-hydroxyphenyl)propanoic acid), and 4-EPhSO4 ((4-ethylphenyl) hydrogen sulfate) (Table 5). All had been previously identified in human plasma [22]. Among them, 4-EPS has garnered increasing attention due to its strong association with autism spectrum disorders [99,100]. Although not detected in mouse brain under baseline conditions, brain levels have been quantified following systemic administration of a high dose of 4-EPS, confirming that it can cross the BBB [101]. Given that 4-EPS is a component of the human blood metabolome [102,103], we anticipated its ability to enter the CSF.

We adjusted our targeted data for age and gender, variables known to affect certain elements of the metabolome [104,105]. Within the FEP group, 12 of 15 participants were receiving APDs while medication data were unavailable for the remaining three. For the purpose of biomarker development, it is important to differentiate between indices that reflect the underlying pathophysiology of the disorder and those that are secondary to treatment. One strategy involves longitudinal sampling from the same individual before and after APD exposure. Another approach compares APD-naïve patients with those undergoing treatment. Additionally, studying patients with non-schizophrenic disorders who are also treated with APDs may yield further insights. We did not adopt such approaches. As a result, we cannot exclude the possibility that APDs may have influenced our findings. Others have suggested that some elements of the CSF metabolome associated with schizophrenia, such as glucose, lactate, glutamine, citrate [23], or certain lipids [24], are affected by APDs. APD effects on Neu5Ac levels have not, to our knowledge, been described. Levels of uric acid in the blood have been reported to be elevated by some APDs [106] and lowered by others [107]. APD effects on CSF uric acid have not been reported. Limited studies suggest that peripheral L-PHE levels are not affected by exposure to APDs [85,108]. We were unable to find any studies examining APD effects on 4-hydroxycinnamic acid in any compartment. Of course, absence of evidence is not evidence of absence.

We did not have data on the dietary patterns of our subjects. Compared to healthy controls, patients with schizophrenia tend to have less nutritious diets [103,109]. Diet has not been shown to affect levels of Neu5Ac, but can affect blood levels of L-PHE; these effects are usually buffered by homeostatic mechanisms and the BBB [81]. Diet can affect uric acid levels in the blood, but the correlation with CSF levels is weak at best [57,58].

Substance use is more prevalent among individuals with schizophrenia [110], and it can influence various metabolomic parameters [111]. In our study, participants with an active substance use disorder (e.g., involving alcohol, cocaine, methamphetamine, or opioids) within two months prior to enrollment were excluded. However, individuals reporting occasional cannabis use or regular tobacco use were included. One healthy control and two FEP participants reported occasional cannabis use (Supplementary Table S3), suggesting that any cannabinoid-related effects on metabolomic outcomes were likely minimal.

As anticipated, nicotine use was significantly more prevalent in the FEP group (Supplementary Table S3). This likely contributed to the elevated cotinine levels and the reduced concentrations of theophylline and theobromine observed in these patients (Table 4), as previously discussed. To our knowledge, the effects of nicotine on the other analytes identified in our exploratory analysis have not been reported.

The primary limitation of our study is the relatively small sample size relative to the large number of analytes measured. Consequently, correction for multiple comparisons imposed a stringent threshold for statistical significance, which none of the analytes met. This limitation must be considered in the context of the unique value of cerebrospinal fluid (CSF), which, compared to blood, more closely reflects the brain’s biochemical environment [20,21] and exhibits less chemical variability [22]. However, the discomfort and non-negligible medical risks associated with lumbar puncture [112] pose significant challenges to participant recruitment for CSF-based studies.

Unsurprisingly, our PubMed search identified relatively few investigations of the CSF metabolome in FEP. One study reported significant differences in CSF levels of glucose, acetate, alanine, citrate, lactate, and glutamine between APD-naïve FEP patients and healthy controls [23]. In studies involving FEP patients with mixed APD exposure, lower GABA levels alongside elevated serotonin and dopamine concentrations have been observed [112,113]. Notably, these analytes do not overlap with those highlighted in a meta-analysis of the CSF metabolome in heterogeneous schizophrenia cohorts [114], nor with the compounds identified in our own study—an outcome that is not unexpected given methodological differences across studies.

## 5. Conclusions

Our exploratory study suggests an association between cerebrospinal fluid (CSF) levels of several analytes and first-episode psychosis (FEP). Although none of the associations survived correction for multiple comparisons—a predictable outcome given the small sample size and the large number of analytes evaluated—several findings warrant further investigation. Our non-targeted analysis suggested an association between FEP and lower CSF levels of Neu5A (Table 4), confirming numerous MRS and brain tissue-based reports [2,28,115]. We also observed lower CSF Neu5Ac levels in the FEP group (Table 4), consistent with post-mortem tissue studies [41]. In contrast, levels of the antioxidant UA were elevated (Table 4). On targeted analysis, FEP was associated with lower levels of L-PHE, a precursor in catecholamine synthesis [13] as well as lower levels of 4-HCA, a bioactive and generally neuroprotective compound, derived from interactions between food intake and the GMB (Figure 6).

We acknowledge that our study did not identify any analytes that met the stringent criteria for biomarker candidacy or withstood correction for multiple comparisons. Additionally, this study was not designed to assess the effects of antipsychotic drug (APD) exposure, and dietary information was not collected. Nevertheless, given the unique value of CSF in reflecting central nervous system biochemistry—and the inherent challenges in obtaining large CSF samples from FEP patients—exploratory studies such as ours remain valuable. They serve as a critical first step in identifying candidate analytes for deeper investigation in larger, hypothesis-driven studies.

This iterative process, guided by prior findings, allows for the refinement of analyte selection and supports the application of less conservative thresholds for multiple comparison correction in future targeted analyses. We have successfully used this approach in previous work, demonstrating that compounds identified as GMB-dependent in the serum of germ-free mice [116] were also affected in serum of a clinical sample exposed to systemic antibiotics [117]. Future studies will determine whether CSF metabolomic findings will stand on their own or will constitute one type of important data within a complex, multimodal biomarker framework [6,118].

## Figures and Tables

**Table 1:** Comparison of the healthy controls (HCs) and first-episode psychosis (FEP) participants. Ordinal variables were compared by unpaired two-sided *t*-test and categorical variable by χ^2^ (*p* < 0.05). Ordinal data are provided as mean + SEM. Abbreviations A—Asian, B—Black, W—White.

Variable	HC	FEP	Significance
Age (y)	23.21 + 1.0	23.47 + 0.96	0.86
Gender M/F	8/6	11/4	0.36
Race A/B/W	1/11/2	1/12/2	0.74
Freezer time (y)	5.95 + 0.35	5.27 + 0.19	0.1

**Table 2:** Features identified by non-targeted analysis.

C18 Chromatography	POS ESI	NEG ESI	Total
Number of Features	6745	451	7196
Number of MS1 Level features matched to MoNA and MSDIAL MSP databases	1339	110	1449

**Table 3:** Comparison of fold change (FC) for n = 1448 features from non-targeted analysis.

FEP vs. HC	Raw *p* < 0.05		Raw *p* > 0.05
Direction of change	↑	↓	
Number of features	7	16	1425

**Table 4:** Fold change of the non-targeted data analyzed by *t*-test, FEP v HC, sorted by raw *p* value.

		FC	log2 (FC)	Raw *p* Value	negLog (Raw *p* Value)
1	N-Acetylneuraminate	0.45425	−1.1384	0.000457	3.3399
2	Phenacylamine Hydrochloride	0.42157	−1.2462	0.00119	2.9246
3	5-(6-acetyloxy-3,5,7-trimethoxy-4-oxochromen-2-yl)-2-methoxyphenyl acetate	0.26672	−1.9066	0.001964	2.7069
4	Modafinil	0.41774	−1.2593	0.003386	2.4704
5	Theobromine.1	0.14956	−2.7412	0.005127	2.2902
6	Indoline	16.123	4.011	0.008778	2.0566
7	Uric acid.1	11.927	3.5762	0.011072	1.9558
8	N-(2,4-Dimethylphenyl)-N-methylimidoformamide	6.2543	2.6448	0.01325	1.8778
9	1,3-Dimethyl-2-imidazolidinon	8.713	3.1232	0.015998	1.7959
10	NCGC00380117-01_C27H41NO4_(7E)-3-Isobutyl-4,5,8,12,12-pentamethyl-3,3a,4,6a,9,10,10a,13a,14,15-decahydro-1H-[1,3]dioxolo[7,8]cycloundeca[1,2-d]isoindole-1,16(2H)-dione	0.26435	−1.9195	0.01624	1.7894
11	PharmaGSID_47259	0.47576	−1.0717	0.019798	1.7034
12	Procaine (Novocaine) HCl	0.46382	−1.1084	0.020015	1.6987
13	Kainic Acid	0.44111	−1.1808	0.022828	1.6415
14	N-2-Hydroxycyclopentyladenosine	0.47246	−1.0817	0.026159	1.5824
15	N-Acetyl-L-aspartic acid.1	0.16743	−2.5783	0.02893	1.5386
16	Theophylline	0.33052	−1.5972	0.032693	1.4855
17	Isoxanthopterin	0.33413	−1.5815	0.037612	1.4247
18	tetradec-5-ynoic acid.1	0.29953	−1.7392	0.038349	1.4162
19	Cotinine	5.1242	2.3573	0.040772	1.3896
20	Theophylline	0.17017	−2.555	0.043098	1.3655
21	Andrachcinidine	2.0015	1.001	0.04501	1.3467
22	2-Hydroxypyridine	2.7989	1.4848	0.045195	1.3449
23	Procaine	0.40754	−1.295	0.046636	1.3313

**Table 5:** Concentrations (nM) of molecules identified in human CSF (nM) by targeted analysis. IDNQ: identified but not quantified. * not previously identified in human CSF. Full data provided in Supplementary Table S4.

Abbreviation	IUPAC Name	CID	CAS	MW	Mean	MED	STDev	% “Non-0” Entries	RT (min)
TAU	2-aminoethanesulfonic acid	1123	107-35-7	125.15	384.99	323.94	189.55	69.0	1.64
CARN	(3R)-3-hydroxy-4-(trimethylazaniumyl)butanoate	10917	541-15-1	161.2	641.07	657.56	356.96	100.0	1.59
IMDZA	3-(1H-imidazol-5-yl)propanoic acid	70630	1074-59-5	140.14	IDNQ			72.4	7.1
3-HPPU	3-(3-hydroxyphenyl)-2-oxopropanoic acid	5318321	4607-41-4	180.16	IDNQ			82.8	1.94
CRE	2-amino-3-methyl-4H-imidazol-5-one	588	60-27-5	113.12	3005.73	1014.08	4852.72	58.6	1.86
XANS	9-[(2R,3R,4S,5R)-3,4-dihydroxy-5-(hydroxymethyl)oxolan-2-yl]-3H-purine-2,6-dione	64959	146-80-5	284.23	57.40	57.40		3.4	6.97
3-HKYN	2-amino-4-(2-amino-3-hydroxyphenyl)-4-oxobutanoic acid	89	484-78-6	224.21	298.07	298.07	272.57	6.9	6.61
HXT	1,7-dihydropurin-6-one	135398638	68-94-0	136.11	166,783.98	199,278.78	106,273.24	100.0	14.3
XA	3,7-dihydropurine-2,6-dione	1188	69-89-6	152.11	80,324.64	89,918.97	49,963.03	96.6	7.4
3,5-DHHCA	3-(3,5-dihydroxyphenyl)propanoic acid	161525	26539-01-5	182.17	726.12	352.90	669.53	37.9	7.16
3,4-DOPAC	2-(3,4-dihydroxyphenyl)acetic acid	547	102-32-9	168.15	1928.23	1589.08	1083.38	51.7	6.99
2,5-DOPAC	2-(2,5-dihydroxyphenyl)acetic acid	780	451-13-8	168.15	IDNQ			55.2	2.18
4-HPPU	3-(4-hydroxyphenyl)-2-oxopropanoic acid	979	156-39-8	180.16	160.14	141.83	99.18	41.4	6.98
3,4-DHCA	(E)-3-(3,4-dihydroxyphenyl)prop-2-enoic acid	689043	331-39-5	180.16	544.50	482.37	352.43	13.8	6.98
3,4-DHPLA *	3-(3,4-dihydroxyphenyl)-2-hydroxypropanoic acid	439435	23028-17-3	198.174	768.68	664.07	338.54	89.7	6.49
SyA	4-hydroxy-3,5-dimethoxybenzoic acid	10742	530-57-4	198.17	IDNQ			96.6	6.5
p-TYR	(2S)-2-amino-3-(4-hydroxyphenyl)propanoic acid	6057	60-18-4	181.19	288.89	296.45	180.88	62.1	2.06
2,4-DHCA	(E)-3-(2,4-dihydroxyphenyl)prop-2-enoic acid	446611	614-86-8	180.16	IDNQ			6.9	6.98
PGN *	benzene-1,3,5-triol	359	108-73-6	126.11	1275.10	1307.20	347.87	62.1	7.01
5-HIAA	2-(5-hydroxy-1H-indol-3-yl)acetic acid	1826	54-16-0	191.18	119.18	102.34	68.34	89.7	7.09
ICA	1H-indol-3-ylmethanol	3712	700-06-1	147.17	IDNQ			17.2	3.15
BA	benzoic acid	243	65-85-0	122.12	4055.59	3856.55	1198.60	41.4	2.5
L-PHE	(2S)-2-Amino-3-phenylpropanoic acid	6140	63-91-2	165.19	61,352.08	69,824.54	21,694.42	100.0	6.46
3-HHA	2-[(3-hydroxybenzoyl)amino]acetic acid	450268	1637-75-8	195.17	5637.01	3256.17	5456.52	100.0	1.7
4,3-HMPPA *	3-(4-hydroxy-3-methoxyphenyl)propanoic acid	14340	1135-23-5	196.2	4647.88	5043.02	2192.95	20.7	7.3
HA	2-benzamidoacetic acid	464	495-69-2	179.17	152.50	137.09	71.06	79.3	7.05
4-HPLA	2-hydroxy-3-(4-hydroxyphenyl)propanoic acid	9378	306-23-0	182.17	2047.23	2023.46	1077.68	100.0	7.15
2,3-DHHCA	3-(2,3-dihydroxyphenyl)propanoic acid	20	3714-73-6	182.17	7.68	6.33	5.99	31.0	7.14
HVA	2-(4-hydroxy-3-methoxyphenyl)acetic acid	1738	306-08-1	182.17	272.91	273.87	76.17	100.0	2.07
2-HBA	2-hydroxybenzoic acid	338	69-72-7	138.12	180.54	47.92	558.66	72.4	1.7
CNG *	2-[[(E)-3-phenylprop-2-enoyl]amino]acetic acid	709625	16534-24-0	205.21	23,947.50	35,344.27	20,400.68	93.1	6.5
ISA (I3XS)	1H-indol-3-yl hydrogen sulfate	10258	487-94-5	213.21	13.49	6.15	17.71	27.6	4.31
3-HPAA	2-(3-hydroxyphenyl)acetic acid	12122	621-37-4	152.15	157.58	115.06	107.09	13.8	7.34
2-HPAA	2-(2-hydroxyphenyl)acetic acid	11970	614-75-5	152.15	694.04	525.22	632.64	24.1	7.3
4-HPAA	2-(4-Hydroxyphenyl)acetic acid	127	156-38-7	152.15	205,728.25	200,232.56	86,633.30	89.7	7.33
IAA	2-(1H-indol-3-yl)acetic acid	802	87-51-4	175.18	1582.66	307.76	4307.78	41.4	10.82
3-HBA	3-hydroxybenzoic acid	7420	99-06-9	138.12	161,373.60	194,364.15	77,760.59	89.7	7.65
3-HCA	3-(3-Hydroxyphenyl)propanoic acid	637541	14755-02-3	164.16	154.87	90.62	156.19	62.1	6.9
4-HCA	(2E)-3-(4-Hydroxyphenyl)prop-2-enoic acid	637542	7400-08-0	164.16	1234.43	1094.32	986.64	44.8	7.59
4-HBA	4-hydroxybenzoic acid	135	99-96-7	138.12	73,092.90	81,531.21	20,714.32	86.2	7.19
IFA	(E)-3-(3-hydroxy-4-methoxyphenyl)prop-2-enoic acid	736186	25522-33-2	194.18	IDNQ			96.6	7.7
PAG	2-[(2-phenylacetyl)amino]acetic acid	68144	500-98-1	193.2	IDNQ			93.1	43.7
RA	(2R)-3-(3,4-dihydroxyphenyl)-2-[(E)-3-(3,4-dihydroxyphenyl)prop-2-enoyl]oxypropanoic acid	5281792	20283-92-5	360.3	IDNQ			24.1	6.9
2-HPPA	3-(2-hydroxyphenyl)propanoic acid	873	495-78-3	166.17	24.59	20.70	9.82	82.8	7.59
2,4-HPPA *	2-(4-hydroxyphenyl)propanoic acid	102526	938-96-5	166.17	74,309.51	75,951.21	7329.52	100.0	7.6
4-HPPA *	3-(4-hydroxyphenyl)propanoic acid	10394	501-97-3	166.17	10,635.33	10,773.43	888.97	100.0	7.84
3-HPPA	3-(3-hydroxyphenyl)propanoic acid	91	621-54-5	166.17	5044.83	4841.94	1227.72	100.0	7.18
PEA-HCl	2-phenylethanamine;hydrochloride	9075	156-28-5	157.64	35.39	29.68	20.03	13.8	7.84
3-IACrA	(E)-3-(1H-indol-3-yl)prop-2-enoic acid	15030923	29953-71-7	187.19	721.67	570.28	617.71	34.5	7.3
PCS	(4-methylphenyl) hydrogen sulfate	4615423	3233-58-7	188.2	302.30	266.79	255.42	27.6	7/7
I3PA	3-(1H-indol-3-yl)propanoic acid	3744	830-96-6	189.21	217.42	212.92	35.39	17.2	3.79
3,5-DHBA	3,5-dihydroxybenzoic acid	7424	99-10-5	154.12	IDNQ			100.0	8.27
4-EPS *	(4-ethylphenyl) hydrogen sulfate	20822573	85734-98-1	202.23	76.45	81.51	46.05	44.8	7.28
N-UNDG *	2-(undecanoylamino)acetic acid	454092	83871-09-4	243.34	185.89	181.04	53.80	34.5	10.49

**Table 6:** Characteristics from the targeted-analysis (as listed in Table 5) that were significantly different between controls and first-episode psychosis (Wilcoxon *p* < 0.1. None reached a false-discovery rate (FDR) < 0.1). 3-hydroxybenzoic acid (3-HBA), 3-hydroxyhippuric acid (3-HHA), 3-hydroxyphenylpropanoic acid (3,3-HPPA), 4-hydroxybenzoic acid (4-HBA), 4-hydroxycinnamic acid (4-HCA), phenylacetylglycine (PAG), phenylalanine (positive mode) (PHE+).

Compound	V	Wilcoxon p	FDR	% Non-Zero Entries
4-HCA	47	0.010482	0.48178	44.8%
3-OHBA	159	0.017844	0.48178	89.7%
PHE(+)	57	0.036742	0.66136	100%
4-HBA	147	0.069659	0.75231	86.2%
3-HPPA	147	0.069659	0.75231	100%
3-HHA	67	0.10231	0.78924	100%
PAG	67	0.10231	0.78924	93.1%

## Data Availability

The original contributions presented in this study are included in the article/Supplementary Materials.

## Supplementary Materials

The following supporting information can be downloaded at: https://www.mdpi.com/article/10.3390/metabo15040275/s1. Table S1. Internal Standards; Table S2. Solvent gradient; Table S3. Participant and CSF sample characteristics; Table S4. All data OPLSDA-VIP; Table S5. Raw data—targeted analysis.
